# *Lactobacillus rhamnosus GG* modifies the metabolome of pathobionts in gnotobiotic mice

**DOI:** 10.1186/s12866-021-02178-2

**Published:** 2021-06-03

**Authors:** Jinhee Kim, Iyshwarya Balasubramanian, Sheila Bandyopadhyay, Ian Nadler, Rajbir Singh, Danielle Harlan, Amanda Bumber, Yuling He, Lee J. Kerkhof, Nan Gao, Xiaoyang Su, Ronaldo P. Ferraris

**Affiliations:** 1 Department of Pharmacology, Physiology and Neurosciences, Medical Science Building, New Jersey Medical School, Rutgers University, Newark, NJ 07103 USA; 2 Department of Biological Sciences, Life Science Center, Rutgers University, Newark, NJ 07102 USA; 3 Comparative Medicine Resources, Rutgers University, Newark, NJ 07103 USA; 4 Department of Medicine, Clinical Academic Building, Robert Wood Johnson Medical School, Rutgers University, New Brunswick, NJ 08901 USA; 5 Present address: Geriatric Endocrinology Division, The First Affiliated Hospital of Guangxi Medical University, Nanning, Guangxi China; 6 Department of Marine and Coastal Sciences, Rutgers University, 71 Dudley Rd, New Brunswick, NJ 08901 USA

**Keywords:** Competitive exclusion, Fecal metabolites, Germ-free mice, Inflammation, Liquid chromatography, mass spectrometry, Microbiota, *Propionibacterium acnes*

## Abstract

**Background:**

*Lactobacillus rhamnosus* GG (LGG) is the most widely used probiotic, but the mechanisms underlying its beneficial effects remain unresolved. Previous studies typically inoculated LGG in hosts with established gut microbiota, limiting the understanding of specific impacts of LGG on host due to numerous interactions among LGG, commensal microbes, and the host. There has been a scarcity of studies that used gnotobiotic animals to elucidate LGG-host interaction, in particular for gaining specific insights about how it modifies the metabolome. To evaluate whether LGG affects the metabolite output of pathobionts, we inoculated with LGG gnotobiotic mice containing *Propionibacterium acnes, Turicibacter sanguinis,* and *Staphylococcus aureus* (PTS).

**Results:**

16S rRNA sequencing of fecal samples by Ion Torrent and MinION platforms showed colonization of germ-free mice by PTS or by PTS plus LGG (LTS). Although the body weights and feeding rates of mice remained similar between PTS and LTS groups, co-associating LGG with PTS led to a pronounced reduction in abundance of *P. acnes* in the gut. Addition of LGG or its secretome inhibited *P. acnes* growth in culture. After optimizing procedures for fecal metabolite extraction and metabolomic liquid chromatography-mass spectrometry analysis, unsupervised and supervised multivariate analyses revealed a distinct separation among fecal metabolites of PTS, LTS, and germ-free groups. Variables-important-in-projection scores showed that LGG colonization robustly diminished guanine, ornitihine, and sorbitol while significantly elevating acetylated amino acids, ribitol, indolelactic acid, and histamine. In addition, carnitine, betaine, and glutamate increased while thymidine, quinic acid and biotin were reduced in both PTS and LTS groups. Furthermore, LGG association reduced intestinal mucosal expression levels of inflammatory cytokines, such as IL-1α, IL-1β and TNF-α.

**Conclusions:**

LGG co-association had a negative impact on colonization of *P. acnes*, and markedly altered the metabolic output and inflammatory response elicited by pathobionts.

**Supplementary Information:**

The online version contains supplementary material available at 10.1186/s12866-021-02178-2.

## Background

*Lactobacillus rhamnosus* GG (LGG) is a Gram-positive facultative anaerobic bacterium of the phylum Firmicutes typically associated, as are many other *Lactobacillus* spp., with the treatment and prevention of intestinal inflammatory disorders [1–3]. Amounts of endogenous *Lactobacillus* spp. are significantly reduced in the intestinal lumen of colitis patients [4]. The LGG strain (ATCC 53103) is the most widely used probiotic species sold under different trademarks [5]. LGG is thought to upregulate the synthesis of anti-inflammatory cytokines and downregulate the production of pro-inflammatory cytokines in a variety of gut-associated disorders [6, 7], but the mechanisms are unknown. The anti-inflammatory characteristics of LGG could be due to the bacterial structure itself as well as to its secreted proteins or metabolites. Heat-killed *Lactobacillus bulgaricus* and cell-surface modified LGG attenuate dextran sulfate sodium (DSS)-induced colitis in mice [8, 9], suggesting that cell surface characteristics underlie at least some of their probiotic properties. Mucus-binding appendages like SpaCBA pili and mucus-binding surface adhesins such as MabA and MBF play a role not only in promoting intestinal colonization by LGG but also in LGG’s anti-inflammatory and immunomodulatory effects [10, 11]. The two secreted LGG proteins, p40 and p75, inhibit apoptosis in mouse intestinal epithelial cells treated with the pro-inflammatory cytokine tumor necrosis factor-alpha (TNF-α), mainly by reinforcing the tight junction barrier, thereby enhancing epithelial integrity [12, 13]. Surprisingly, there has been a scarcity of studies investigating LGG-associated gut microbial metabolites. This is unfortunate, as Lactobacillus spp. may produce metabolites that can act as anti-inflammatory mediators. For example, Aryl Hydrocarbon Receptor (AHR) ligands from dietary and Lactobacilli-metabolized tryptophan utilized AHR signaling to mediate their beneficial effects, including exclusion of pathobiont species and mucosal protection from inflammation [14, 15].

A large number of studies of LGG and other *Lactobacillus* spp. incorporated bacterium in feeds or drinking water [1–3, 16–19]. In these studies, a *Lactobacillus* species was typically inoculated in a host that housed an established commensal microbiota [20]. Results from these studies reflected an outcome from interactions among *Lactobacillus,* numerous existing microbes, and the host, with limited insight on the specific impact of *Lactobacillus*. In contrast, more recent studies used *Lactobacillus* mono-association in gnotobiotic animals to investigate *Lactobacillus*-host interactions [21–24]. Although these mono-association approaches appeared to be more specific in elucidating *Lactobacillus*-host interactions, the mucosal environment completely lacked the presence of other microbial components, thereby limiting the discovery of physiological insights. In our experiment, we analyzed the effect of a *Lactobacillus* probiotic on gnotobiotic mice housing common commensal bacteria typically ingested by humans along with food which, more importantly, also cause gastrointestinal illnesses under certain conditions [25, 26].

*Propionibacterium acnes* is a slow-growing facultative anaerobic Gram-positive bacterium found in the gut and skin, and is considered to be a pathobiont as it plays a pivotal role in promoting gastrointestinal disorder and acne, respectively, during dysbiosis [27–29]. While relatively benign, *P. acnes* was an important opportunistic pathogen causing implant-associated infections, and thought to initiate chronic lymphocytic gastritis [30]. Patients with a high abundance of *Helicobacter pylori*, *P. acnes* and *P. copri* were at a higher risk of developing gastric cancer [31]. *Turicibacter sanguinis* is a strictly anaerobic, Gram-positive, rod-shaped commensal bacterium found in the gut and feces of many animals [32, 33]. *T. sanguinis* was associated with high levels of an antimicrobial fatty acid, butyrate, and of a proinflammatory cytokine TNF in the gut [34, 35] and was recently discovered to express a sodium symporter protein that absorbed the neurotransmitter serotonin (5-hydroxytryptamine) [36]. Interestingly, it is depleted in dogs with inflammatory bowel disease [33]. In hosts associated with *T. sanguinis*, the bacteria affected lipid and steroid metabolism, with corresponding reductions in host systemic triglyceride levels and adipocyte size [36]. Commonly responsible for numerous cases of food poisoning [37], *Staphylococcus aureus* is a facultative anaerobic Gram-positive, round-shaped, commensal bacterium that is frequently found in the upper respiratory tract and the skin. The human intestinal tract is commonly colonized by *S. aureus* and it is unclear how this bacteria becomes virulent as to cause serious outbreaks of gastrointestinal infections [38, 39]. *S. aureus* does produce a heat-stable enterotoxin which, within hours after bacterial ingestion, causes severe intestinal inflammation resulting in abdominal cramping and diarrhea [40].

In this study, we tested the hypothesis that LGG co-association modifies not only the metabolome but also the mucosal inflammatory response to a pathobiont community. We compared mice with *P. acnes, T. sanguinis* and *S. aureus* (PTS) to mice additionally inoculated with LGG (LTS). We demonstrated robust modulatory effects of LGG on colonization by pathobiont bacteria, their metabolome output, and host intestinal inflammation.

## Results

### Fecal metabolite extraction normalization minimized inter-sample variations

Fecal metabolomic analysis has been increasingly used to identify and characterize host-microbe interaction associated with metabolite profiles. However, a challenge has been the large inter-sample variabilities that may be attributed to procedural and other factors unrelated to host or microbial pathways of interest. These include different extraction methods, variable amounts of fecal material, and batch-to-batch sampling discrepancies. Previous studies have shown that extraction of mouse feces and subsequent normalization played a role in determining the quality of analysis [41, 42]. The efficacies of various extraction protocols have been compared in earlier studies [43, 44]. However, even when a unified extraction methodology [43] was followed, we noted considerable variations across samples. We thus designed a series of experiments to gauge the amount of variabilities attributable to sample sizes and collection batches, with the overarching goal of developing an optimal LC-MS method, which, upon minimizing non-biological variabilities, allowed for a robust and reproducible identification of metabolic features characteristic of host-microbe interaction.

**Fig. 1:**
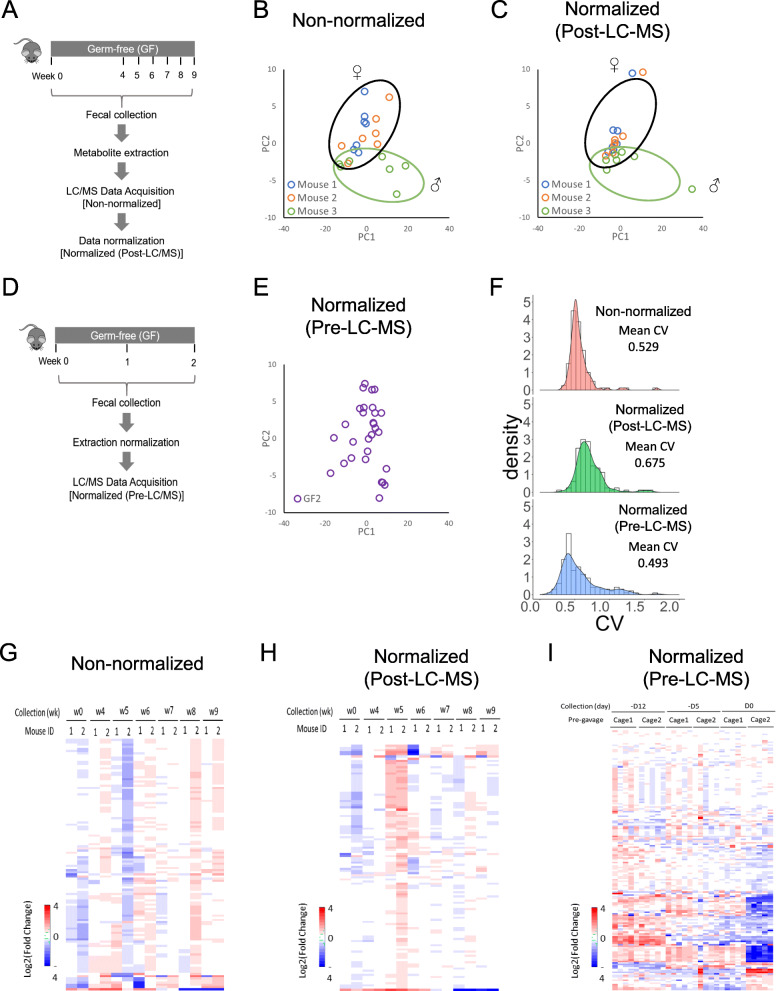
Comparison of different LC-MS normalization procedures. **a** Mice were fed for 9 weeks, and feces were sampled in weeks 0, 4, 5, 6, 7, 8 and 9. Mice remained germ-free (GF) throughout. Equal volumes of extraction buffer were added to all fecal samples regardless of the fecal weight. After LC-MS analysis, the data either remained non-normalized, or were normalized to fecal weight (Post-LC-MS). **b** PCA analysis of the non-normalized (to fecal weight) LC-MS metabolite data. **c** PCA analysis of the LC-MS metabolite data normalized to fecal weight. **d** Feces of GF mice were collected three times over two weeks. Prior to LC-MS analysis, they were weighed and the volume of the extraction buffer was adjusted to correspond to the weight of the feces (Pre-LC-MS). **e** PCA analysis of the fecal metabolites in GF mice used in (**d**). **f** Density plot of the CV (coefficient of variation) under different normalization conditions: non-normalized, post-LC-MS normalized, and pre-LC-MS normalized. **g** Heatmap of non-normalized LC-MS analysis of fecal metabolites from (**a**). **h** Heatmap of post-LC-MS-normalized data of fecal metabolites from (**a**). **i** Heatmap of pre-LC-MS normalized data of fecal metabolites from (**d**)

We used fecal samples from GF mice to minimize the biological variabilities prominently caused by the versatile microbial community. Age-matched GF C57/BL6 mice were housed in individual cages (*n =* 3) in a gnotobiotic facility and fed with an autoclavable diet throughout the study (Supplemental Table 1A). Over a period of 9 weeks, 7 batches of fecal samples were longitudinally collected from each mouse, weighted, and snap-frozen for storage. We chose to use LC-MS due to higher detection sensitivity and broader coverage of polar metabolites compared to other metabolomics tools such as nuclear magnetic resonance [45]. Therefore, global metabolomic profile was determined by targeted LC-MS in both positive and negative ion modes (Fig. 1a).

A batch of 21 fecal samples (7 samples per mouse) of different sizes were extracted and analyzed by LC-MS. Without normalization of the abundance of metabolites against fecal weight, PCA did not identify any noticeable clustering, even among samples collected from the same animal (Fig. 1b), illustrating the considerably large sampling variabilities. We then applied an universal approach of sampling normalization developed by Xu et al. [46] in which metabolite concentration was normalized to the weight of the respective fecal sample at different collection days after LC-MS analysis (referred to as post-LC-MS normalization). The normalized PCA plot showed that 18 out of 21 fecal extracts exhibited tight clustering among samples collected from the same animal at a different collection day (Fig. 1c). The 3 outliers (one from each mouse) were samples obtained on the same collection day and their average fecal weight was only 15–40% of the samples from the other 6 sampling days. This suggested that the fecal sample weight contributed to variations in metabolic profile. Otherwise, the fecal metabolic profile of samples from male and female mice post LC-MS normalization (compare mouse 3 with 1 and 2, Fig. 1c) was the same. We thus performed our subsequent experiments using only female GF mice.

To determine whether accounting for sample weight differences during metabolite extraction would reduce sample-to-sample variations, we longitudinally collected fecal samples from 10 female GF mice housed in 2 separate cages (*n =* 5 per cage). We normalized the extraction buffer volumes to each sample’s weight during metabolite extraction (referred to as pre-LC-MS normalization) (Fig. 1d, e), resulting in extracts having concentrations around 13.33 mg feces/ml. A total of 30 GF fecal samples were analyzed by LC-MS (3 longitudinal collections per mouse, *n =* 10). Density plots of coefficient of variation (CV) were generated to visualize and compare the distribution in non-normalized, post-LC-MS, and pre-LC-MS analyses (Fig. 1f). The pre-LC-MS normalization showed the lowest mean CV of 0.493 compared to non-normalization (mean CV of 0.529) and post-LC-MS normalization (mean CV of 0.675), suggesting a greatly reduced variance across all samples. Heat map analysis of the distribution of metabolites non-normalized (Fig. 1g), post-LC-MS normalized (Fig. 1h), and pre-LC-MS normalized (Fig. 1i) conditions illustrated a reduced variance across samples under pre-LC-MS normalization. Thus, metabolite extraction normalized to fecal weight was proven to be an optimal approach in terms of suppressing variations in metabolomic analysis by LC-MS.

### LGG displaces pathobiont bacteria

**Fig. 2:**
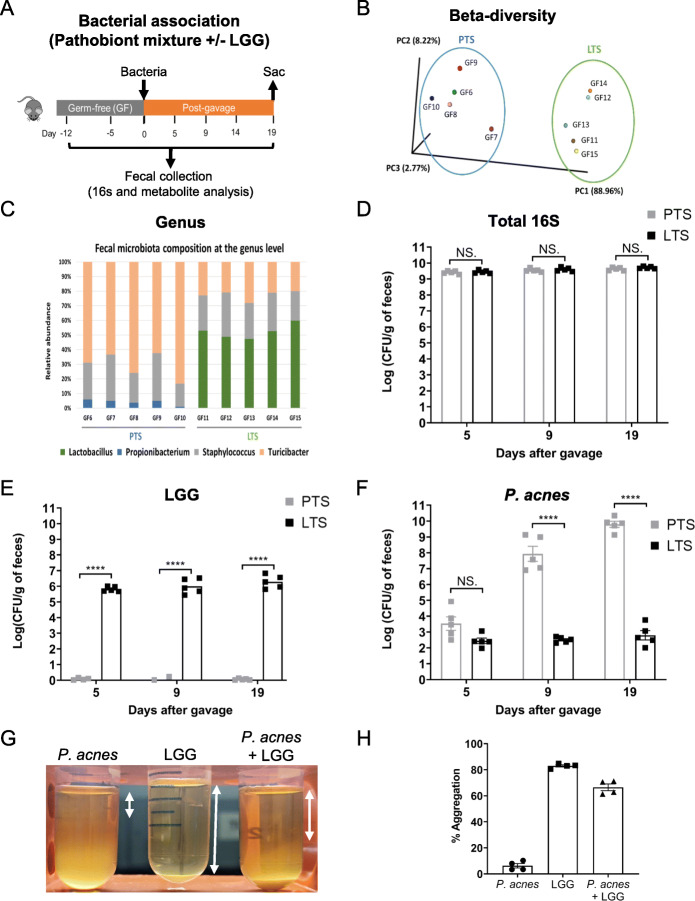
LGG modifies bacterial and fecal metabolite composition of PTS. **a** Schematic diagram of the experimental design. Mice remained GF for 12 days, and on day 0, bacteria (PTS or LTS) were gavaged then feces were sampled on days 0 (before gavage), 5, 9, 14 and 19. Metabolites from feces were extracted using the pre-LC-MS procedure outlined above, then subjected to 16S metagenomic sequencing and metabolomics. **b** Principal coordinate analysis (PCoA) indicating beta diversity of bacterial communities in PTS and LTS mice after gavage. Each point refers to fecal samples in specific mice, with GF6 to 10 mice in the PTS cage, and GF11 to 15 in the LTS cage. **c** Microbiota composition (relative OTU abundance) at the genus level of feces collected 14th day postgavage from PTS and LTS mice. **d** Abundance of 16S rRNA (**d**), LGG (**e**), and *P. acnes* (**f**) in the feces of PTS and LTS mice (*n* = 5 in each group) 5, 9, and 19 days after gavage. **g** Images of aggregation of *P. acnes* alone, LGG alone, and *P. acnes* and LGG in medium after 48 h of incubation. Percentage of autoaggregation of *P. acnes* and LGG and coaggregation of *P. acnes* with LGG (**h**) after 48 h of incubation

Based on the aforementioned optimization of extraction and analysis, we next determined the impact of LGG on the metabolomic output of *P. acnes, T. sanguinis,* and *S. aureus.* Fecal samples were longitudinally collected before and after bacterial association by gavage (Fig. 2a and Supplemental Fig. 1A) from 10 GF mice (*n =* 5 per cage) fed a double-irradiated diet (Supplemental Table 1B). One cage of GF mice (*n =* 5) contained mostly PTS, while the other cage of GF mice was colonized with LGG in addition to the PTS (referred to as “LTS” group). 16S rRNA sequencing by Ion Torrent showed mice either had PTS and LTS groups (Fig. 2b-c and Supplemental Fig. 2A-D). The body weights and feeding rates of mice remained similar pre- and post-gavage between PTS and LTS groups indicating that fecal metabolite quantities between groups were not confounded by these factors (Supplemental Figs. 1B-C).

Quantitative PCR with 16S rRNA-specific primers revealed similar levels of bacterial colonization in PTS and LTS mice (Fig. 2d). Using LGG-specific primers, successful colonization was confirmed in LTS mice (Fig. 2e). There was no LGG in PTS mice. *P. acnes* levels increased > ~ 400,000-fold over 2 weeks post-gavage in PTS mice but were only present in negligible numbers in LTS mice (Fig. 2f). Addition of LGG clearly led to a pronounced reduction in the relative abundances of *Turicibacter* and *Propionibacterium* (Fig. 2c and Supplemental Fig. 2A-D), indicating an LGG-mediated exclusion of these bacteria. We confirmed the inhibitory effect of LGG on *P. acnes* in vitro, as this species was virtually eliminated by LGG whereas *T. sanguinis* and *S. aureus* were not. LGG interacted with *P. acnes* leading to a 66.5% coaggregation, compared to 6.23% auto-aggregation by *P. acne*s (Fig. 2g-h).

### LGG modified fecal metabolomic output of pathobionts

**Fig. 3:**
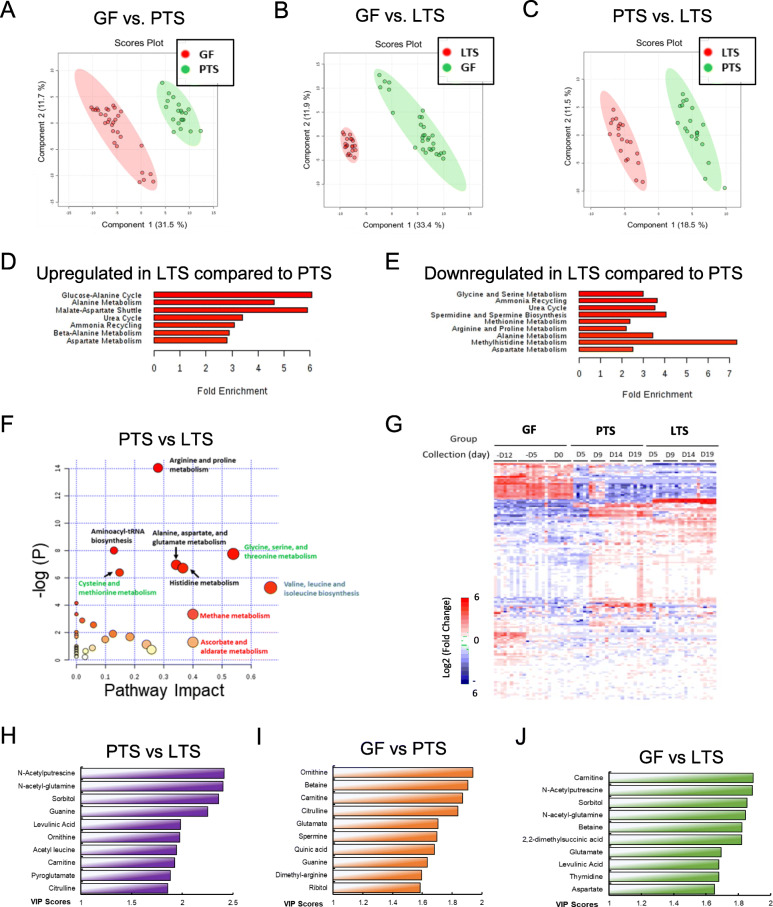
Metabolomic signatures of feces from GF, PTS and LTS mice. Partial least squares discriminant analysis (PLS-DA) scores plots of fecal metabolites from GF and PTS mice (**a**) from GF and LTS mice (**b**) and from PTS and LTS mice (**c**). There are 30 samples for GF, from 3 sampling days and 2 cages, *n =* 5 per cage. There are 20 samples each for PTS and LTS, from 4 sampling days and one cage. **d** Enrichment of metabolic pathways and pathway analysis of t-test significant upregulated metabolites using pathway dataset and pathway analysis of LTS compared to PTS. **e** Enrichment and pathway analysis of t-test significant downregulated metabolites using pathway dataset and pathway analysis of LTS compared to PTS. **f** Metabolic pathway significance plotted against pathway impact of metabolites from PTS and LTS mice. The larger the diameter of the symbols, the greater the impact. **g** Heat map of metabolites illustrating changes in the metabolomic profiles of five GF mice after LTS and PTS gavage. Note the remarkable wide-ranging changes when GF mice were gavaged with bacteria, and the more specific changes between PTS and LTS mice. The top 10 metabolites selected on the basis of VIP scores after comparative analysis of feces from PTS and LTS (**h**) GF and PTS (**i**) and of GF and LTS (**j**) mice. The ordinate represents the VIP scores of metabolites regardless of ionization mode, and of up or down regulation (Supplemental Table 3A to C). If both positive and negative ionization modes of a single metabolite had high VIP scores, only the higher VIP score was used

We then used LC-MS to examine fecal metabolites of PTS mice. Both unsupervised multivariate PCA (Supplemental Fig. 3) and supervised PLS-DA demonstrated a distinct separation between metabolites of PTS mice and pre-gavage GF mice (Fig. 3a), between those of LTS and GF mice (Fig. 3b), and between those of PTS and LTS mice (Fig. 3c). These data when analyzed further to determine significant variable’s important in the projection (VIP) scores as discussed in the next section below, suggested a global alteration of metabolic output by PTS after LGG is administered in vivo.

By performing enrichment and pathway analysis using MetaboAnalyst 4.0 [47], we examined the metabolic pathways altered by PTS as well as those impacted by LGG co-association. Compared to pre-gavage GF mice, PTS significantly upregulated ammonia recycling, glycine and serine metabolism, urea cycle, methylhistidine metabolism, arginine and proline metabolism, spermidine and spermine biosynthesis, as well as aspartate metabolism, while downregulating alanine metabolism, glucose-alanine cycle, urea cycle, purine metabolism, as well as glycine and serine metabolism. Compared to PTS, addition of LGG significantly upregulated 7 and downregulated 9 metabolic pathways (Fig. 3d, e, f). Several pathways are both upregulated and downregulated, suggesting that individual reactions within these pathways can be independently regulated to increase or decrease with LTS conditions.

### LGG alters metabolic prolife of PTS mice

The alteration of metabolomic profile by LGG was strongly corroborated by the heat map visualization of polar metabolites (Fig. 3g). VIP analysis revealed 43 unique metabolites that had > 1.2 VIP scores in the GF vs. PTS comparison, 47 in the GF vs. LTS comparison, and 33 in PTS vs. LTS comparison (metabolites that appeared in both positive and negative ionization modes were counted only once). The selection of VIP cut-off score of > 1.2 was based on discriminatory power when VIP > 1 was considered influential [48]. By directly comparing PTS to LTS, we identified the top 10 metabolites with the highest VIP scores (Fig. 3h) that were either up-regulated or down-regulated in LTS (Supplemental Table 3A). We also identified the top 10 metabolites most significantly correlated with PTS (Fig. 3i, Supplemental Table 3B) or with LTS (Fig. 3j, Supplemental Table 3C) colonization of a GF condition. Metabolites that exhibit high (> 1.2) VIP scores fall into four patterns: those that increase in both PTS and LTS, diminish in both PTS and LTS, decrease mainly in LTS, and increase primarily in LTS (see Supplemental Table 3A,B,C). An important summary of the comparisons of VIP scores between GF and PTS, between GF and LTS, as well as between LTS and PTS, is shown (Supplemental Table 3D).

#### Metabolites upregulated with PTS and LTS

**Fig. 4:**
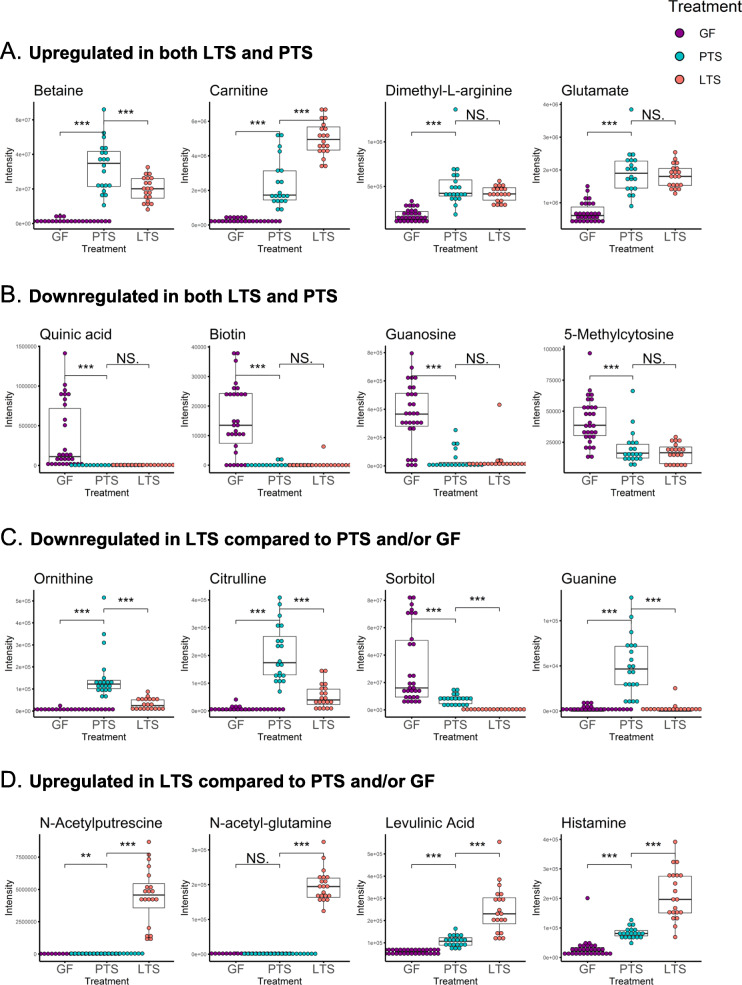
Patterns of regulation of identified metabolites in GF, PTS and LTS mice. **a** Box-whisker plots of representative upregulated metabolites in LTS and PTS mice compared to GF. **b** Box-whisker plots of representative downregulated metabolites in LTS and PTS mice compared to GF. **c** Box-whisker plots of representative downregulated metabolites in LTS compared to PTS and/or GF mice. **d** Box-whisker plots of representative upregulated metabolites in LTS compared to PTS and/or GF mice. * *P* < 0.05; ** *P* < 0.025; *** *P* < 0.001; NS = not significant, *P* > 0.05

Fecal metabolites found in high levels in both PTS and LTS groups may arise from bacterial synthesis of dietary precursors, bacterial modification of dietary metabolites, or from intestinal excretion by the host into the lumen. These include the amino acids betaine, carnitine and glutamate as well as the amino acid derivative dimethyl-L-arginine, all of which exhibit the highest VIP ranks and scores in all three comparisons of PTS and GF, of LTS and GF, as well as of PTS and LTS (Supplemental Tables 3A-D). These metabolites increase dramatically in fecal levels in both PTS and LTS compared with the pre-gavage GF condition (Fig. 4a). Other physiologically important metabolites found in high levels in the feces of PTS and LTS mice were the amino acids aspartate and histidine, vitamin B_3_ (nicotinate), and the polyamine spermine, which have VIP scores of 1.5–1.7 in both PTS and LTS (Supplemental Table 3A-D). Carnitine, betaine, glutamate, N-acetyl-glutamate, histidine, nicotinate, dimethyl-L-arginine and spermine were also present in the diet, but clearly increased markedly in fecal levels after colonization by both the bacterial groups (Fig. 4a). N-acetyl-glutamate was not found in the diet and is likely a major product of bacterial or host metabolism. N-acetyl-glutamate synthase, known to synthesize N-acetylglutamate from glutamate and acetyl-CoA, is expressed by many bacteria and the mammalian small intestine [49].

#### Metabolites downregulated with PTS and LTS colonization

Fecal metabolites found in low concentrations in both PTS and LTS groups when compared to pre-gavage conditions might be reduced by bacterial catabolism (Fig. 4b). The nucleosides thymidine, inosine and guanosine, the nucleotide derivative 5-methylcytosine, the vitamin B_7_ biotin and the plant polyol quinic acid (VIP score ~ 1.4 to 1.7) were highly downregulated in PTS and LTS groups compared to pre-gavage. Quinic acid is abundant in plants, but could also be produced from corn starch which constitutes ~ 55% of our purified diet, by irradiation [50] (Supplemental Table 1B). Quinic acid is readily metabolized by the gut microbiota [51], explaining reduced concentrations in the feces of PTS and LTS groups relative to pre-gavage.

#### LGG diminished PTS-derived metabolites

Ornithine, citrulline, guanine (Fig. 4c) and isoleucine (not shown) were synthesized abundantly by PTS then were strongly catabolized by LGG. Sorbitol and pyruvate were catabolized from the diet by both PTS and LTS, but LGG greatly enhanced this catabolic activity (Fig. 4c; Supplemental Table 3D).

#### Metabolites uniquely upregulated by LGG colonization

Levels of N-acetyl-putrescine, N-acetyl-glutamine (Fig. 4d) and indolelactic acid (not shown) were similar between PTS and pre-gavage, suggesting none of the PTS bacteria were able to synthesize these metabolites (Supplemental Tables 3A-D). Their levels increased markedly in LTS mice, resulting in high VIP scores of > 1.7. This suggested their collective importance to the distinctive metabolome of LTS. Levulinic acid, a by-product of the gut neurotransmitters histamine and cellulose, (Fig. 4d) as well as the fatty acid 2,2-dimethylsuccinic acid (not shown) also had high VIP scores in LTS group (Supplemental Table 3A-D). These fecal metabolites increased modestly in PTS, but were highly upregulated in LTS mice, suggesting that LGG colonization markedly enhanced their production compared to PTS. Other fecal metabolites found in high levels in LTS compared to PTS and pre-gavage were amino acid derivatives acetyl leucine and pyroglutamate, the pentose alcohol ribitol, and purine derivative hypoxanthine.

### Unidentified metabolite features in PTS and LTS mice

**Fig. 5:**
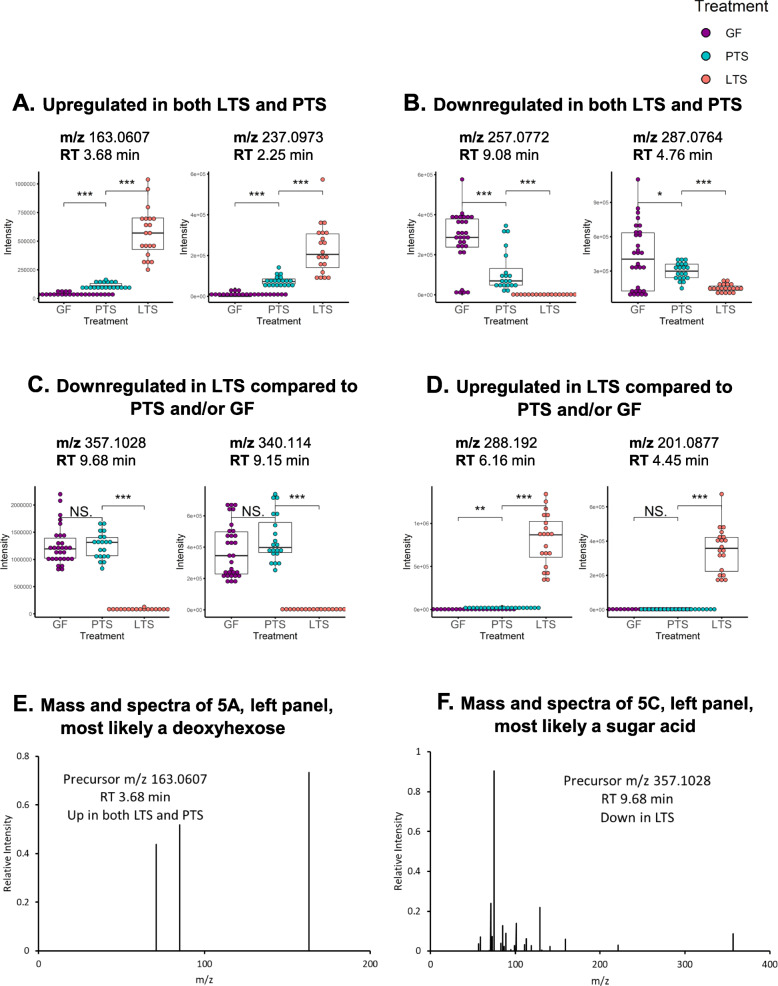
Patterns of regulation of unidentified metabolites in GF, PTS and LTS mice. Box-whisker plots of representative upregulated, unidentified metabolites in LTS and PTS mice compared to GF (**a**), of downregulated metabolites in LTS and PTS mice compared to GF (**b**), of downregulated metabolites in LTS compared to PTS and/or GF mice (**c**), and of upregulated metabolites in LTS compared to PTS and/or GF mice (**d**). Mass spectra of selected unidentified metabolites (**e**, **f**)

In addition to the targeted metabolomics data analysis, we performed untargeted analysis to reveal more metabolomics features that were impacted by bacterial colonization. Similar to the results from the targeted analysis, we found features that fall into four categories as described earlier (Fig. 5a-d). The exact identities of these metabolites were not known. However, based on the accurate mass and MS^2^ spectra (Supplemental Fig. 4, Supplemental Table 4), we suspect one metabolite ([M-H]^−^ m/z 163.0607, retention time 3.68 min) that was upregulated in both LTS and PTS groups to be deoxy-hexose. Another metabolite ([M-H]^−^ m/z 357.1028, retention time 9.68 min) that is downregulated in LTS group could be a sugar acid C_12_H_22_O_12_ (Fig. 5e-f, Supplemental Table 4). These results suggested that carbohydrate metabolism was significantly impacted by the LTS and PTS colonization.

### LGG suppressed some inflammatory features of the pathobionts

**Fig. 6:**
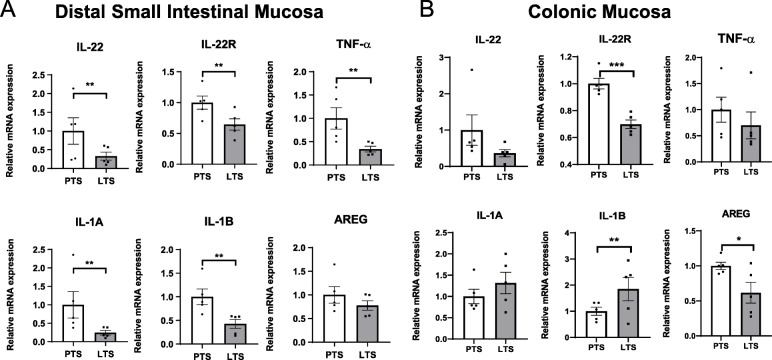
LGG diminishes inflammatory response to pathobiont association. Relative mRNA expression of intestinal immune-related genes in distal intestinal (**a**) and colonic (**b**) mucosa in GF mice associated PTS or LTS (mean ± SEM (*n =* 5). AREG = amphiregulin. * *P* < 0.05, ** *P* < 0.01

As PTS bacteria were implicated in gastrointestinal pathogenesis, we next examined the impact of LGG on PTS-elicited mucosal immune responses by evaluating various inflammation-related genes in proximal and distal small intestines and colons. Interestingly, the expression levels of IL-22 and IL-22R were both significantly down-regulated in the mucosa of all intestinal regions in LTS group (Fig. 6a and b, Supplemental Fig. 5), suggesting LGG-mediated suppression of IL-22 signaling.

The pro-inflammatory cytokine, TNF-α, was significantly down regulated in the distal and proximal small intestinal mucosa of LTS mice (*P* < 0.05, Fig. 6a, Supplemental Fig. 5). Likewise, IL-1α and IL-1β showed similar reductions only in small intestines of LTS mice (*P <* 0.05) (Fig. 6a and b, Supplemental Fig. 5). In contrast, Amphiregulin which was reported to increase following intestinal tissue injury [52], was significantly reduced in colonic mucosa of LTS mice (Fig. 6, Supplemental Fig. 5)*.*

## Discussion

### Potential role of specific LGG-upregulated metabolites in inflammatory response

Competitive exclusion of pathogens is thought to be one of the most important benefits of probiotics, but the mechanisms of exclusion are unknown. While we cannot discount other mechanisms like competition for nutrients and for binding sites, the remarkable change in fecal metabolome elicited by LGG suggests that secretion of certain metabolites may create a hostile luminal ecology for the pathobionts (*Section 4*, Supplemental Table 3D). Interestingly, here we found that LGG increases levels of levulinic acid, a potent anti-bacterial agent targeting mainly pathobionts as it inactivates *Salmonella* and verotoxigenic *E. coli* O157:H7 [53]. Levulinic acid is derived from bacteria-mediated degradation of cellulose as well as related carbohydrates and interestingly, can be made from fructose as well as HCl. It is produced by *L. bulgaricus* in culture, is relatively nontoxic to host cells, and even supports growth of various *Lactobacillus* spp. Levulinic acid also is often used as a component in anti-inflammatory medications, and a derivative, 5-Aminolevulinic acid, reduced expression of pro-inflammatory cytokines TNF-α, cyclooxygenase2, IL-1β, and IL-6 as well as of iNOS and nitric oxide (NO) expression in the lipopolysaccharide-stimulated macrophage cell line RAW264.7 [54]. These findings on cytokine expression are similar to what we observed in the ileum of LGG-treated mice which showed remarkable reductions in TNFα and IL1β expression.

Other LGG-upregulated fecal metabolites also seem to have anti-inflammatory effects. The GI tract has many histamine receptors mediating the numerous functions of histamine, the most important of which are to regulate gastric acid secretion and intestinal inflammation [55]. Histamine is produced from L-histidine via histidine decarboxylase by some fermentative bacteria including Lactobacilli, and in fact, LGG has been shown to secrete histamine, explaining our findings of upregulation in LTS mice [56]. Intestinal luminal conversion of histidine to histamine by the probiotic *L. reuteri* activates the mouse histamine receptor H2R which subsequently results in suppression of IL-6 and IL-1β expression as well as of acute inflammation in TNBS-induced colitis [56], and suppression of inflammatory TNF signaling via modulation of PKA and ERK pathways [57].

The AHR ligand indolelactic acid is specifically produced by several *Lactobacillus* spp., from tryptophan [58], and has been shown to interact with the AHR of a human immature small intestinal cell line to prevent transcription of the inflammatory cytokine IL-8 [59], to prevent production of IL-6 in irradiated human keratinocytes [60], and even to scavenge free radicals [61]. Indolelactic acid produced by *Lactobacillus* spp. also seems to play an important role in regulating differentiation of T cells in the gut. CD4^+^CD8αα^+^ double-positive intraepithelial lymphocytes are a recently discovered class of intestinal T cells missing from GF mice but are believed to take part in immune tolerance. *L. reuteri*-synthesized indolelactic acid via the AHR receptor reprograms CD4^+^-T cells to differentiate into immunoregulatory CD4^+^CD8αα^+^-T cells [15]. Similarly, *L. murinus*-synthesized indolelactic acid prevents salt-sensitive hypertension by reducing levels of pro-inflammatory IL-17A-producing CD4^+^ T_h_17 cells [62].

The link between the other metabolites upregulated in LTS and inflammation is less clear. There are numerous acetylated amino acids upregulated in the LTS group. Acetylation of amino acids seems a unique contribution of and may be a biological signature of LGG. In many bacteria, N-acetyltransferases move an acetyl group from acetyl-CoA to a large array of substrates, including naturally occurring polyamines like putrescine. Per KEGG pathway, this enzyme family is expressed in different strains of *L. rhamnosus*. The N-acetylated form of putrescine is a metabolite of many bacteria, including *Lactobacillus* spp. A high level of N-acetylputrescine was found in the feces of patients with ulcerative colitis and IBS [63]. N-acetyl-glutamine is a psychostimulant, nootropic, and anti-ulcer agent. *L. plantarum* increases production of N-acetylated amino acids, including N-acetyl-glutamine, to enhance *Drosophila* growth, thereby demonstrating an adaptive process by which a symbiotic bacterial strain increases its benefit to its animal host [64]. Ribitol, a pentose alcohol, is metabolized to teicholic acids used in the cell walls of Gram-positive bacteria.

### Potential effects of LGG-downregulated metabolites

Many bacteria can synthesize arginine from glutamate via citrulline and ornithine [65] which is the precursor of polyamines like spermine also present in high concentrations in the feces of PTS mice. Arginine and ornithine are the main metabolic precursors of citrulline, and the conversion of arginine to citrulline releases NO. Ornithine, citrulline and arginine levels can indirectly but markedly affect intestinal function mainly through perturbations in levels of nitric oxide which regulates the permeability of the endothelium in the intestinal vasculature, and of epithelial cells in the mucosa [66]. In humans, high plasma levels of citrulline, as exhibited in our PTS mice, denote injury to the intestinal mucosal barrier and are correlated with high plasma levels of IL-8 [67]. Unfortunately, there has been no study on the effect of luminal citrulline on the mucosal barrier.

Sorbitol can be made from glucose by aldose reductase [68]. It seems to be consumed modestly by PTS, but association with LGG effectively removed sorbitol from the feces, suggesting that LGG is a much more potent consumer of sorbitol (Fig. 4c). In fact, sorbitol has been shown to be utilized by many strains of *L. rhamnosus* [69], including LGG [70]. LGG expresses GutR, a factor that regulates the enzyme unit catabolizing sorbitol (http://regprecise.lbl.gov/RegPrecise/genome.jsp?genome_id=506). Functionally, sorbitol is a sugar alcohol that works as a laxative by drawing water into the large intestine, thereby stimulating intestinal motility. Only a small amount of sorbitol is absorbed in the small intestine, and most enters the colon to elicit gastrointestinal effects. Patients with untreated celiac disease can present sorbitol malabsorption, which is an important cause for persisting disease symptoms. Overdose or improper use of sorbitol may cause severe gastrointestinal complications such as perforated colonic ulcers, ischemic colitis, colonic necrosis, and bleeding. These can likely be minimized by the intake of the probiotic LGG.

### Other significant VIP fecal metabolites

Numerous Gram-positive and Gram-negative bacteria, including *Lactobacillus* spp. and other gut bacteria, synthesize both carnitine and betaine not only for metabolism but also for osmoprotection and bile tolerance which enables colonization of the gut lumen [71, 72]. Carnitine synthesis pathways are very highly conserved and are found in most fungi, bacteria, plants, and animals where carnitine shuttles long-chain fatty acids into the mitochondria so these can be oxidized to produce energy, and then transports toxic compounds out of the mitochondria to prevent their accumulation.

Many bacteria including *Lactobacillus* spp. can produce glutamate by fermentation [73]. Glutamate is important for two reasons. First, glutamate is a precursor to the important neurotransmitter γ-aminobutyric acid (GABA). Both gut bacteria and host cells can synthesize GABA through the decarboxylation of glutamate by glutamate decarboxylase whose encoding gene is present in gut microbiota. Luminal GABA from bacteria is then absorbed by the host small intestine for systemic use elsewhere in the body. Second, glutamate, along with glutamine, is a very important metabolic energy source of the intestinal mucosa. Luminal glutamate from dietary and bacterial sources is typically rapidly absorbed by the small intestine via the transporter EAAT3.

Nicotinate is a water-soluble B3 vitamin found in various animal and plant tissues. It is required by the body for the formation of coenzymes NAD+ and NADP+. It is not surprising that fecal levels of nicotinate are high in PTS and LTS mice, as the majority (~ 60%) of species in the gut microbiota, including members of all five major bacterial phyla, contain nicotinate biosynthesis pathways [74]. Nicotinate and members of the niacin family can be absorbed by the sodium-dependent monocarboxylate transporter SMCT1 in the host intestine. The high fecal glutamate and nicotinate levels in both PTS and LTS mice are likely due to the absence of EAAT3 and SMTC1 in the colon [75].

Although it accumulates in the feces, little is known about the synthesis of dimethyl-L-arginine. Its levels are physiologically important because it is an endogenous inhibitor of nitric oxide which regulates intestinal epithelial and endothelial permeability [66]. Interestingly, dimethyl-L-arginine concentrations increase in the serum of IBD patients [76]. Our novel findings here are likely very important as the intestinal capillaries of patients with IBD show microvascular endothelial dysfunction.

Because biotin, like many other “B” vitamins, is produced by a large number of gut microbiota species, it is surprising that this vitamin’s concentration is reduced in the feces of PTS and LTS mice. The most likely explanation is that, unlike glutamate and nicotinate as mentioned above, biotin is not only more rapidly absorbed by the host, but its transporter, SMTV, is also expressed in the colon [77]. *Bacteroides fragilis*, *L. helveticus* and *Campylobacter coli* are common gut bacteria that can biosynthesize biotin [74, 78]. Thymidine, inosine and guanosine are nucleosides that, along with other nucleosides, nucleotides and nitrogenous bases, are assimilated rapidly by *Lactobacillus* spp. and other bacteria.

### LGG suppresses mucosal inflammatory response to pathobiont

Proinflammatory cytokines like IL-1α, IL-1β and TNF-α seem to be consistently downregulated by LGG in both regions of the small intestine, but not in the colon. This is consistent with several reports that many Lactobacilli species reduce IL-1β and TNF-α levels in the presence of pathogenic bacteria [2, 7, 79]. *Lactobacillus* spp. are primarily located in the small intestine where they exert their influence on the gut microbiota and on the host mucosa [80]. We and others have shown that LGG secretes histamine which many Lactobacilli use to activate the histamine receptor H2R and inhibit TNF signaling and IL-1β expression [56, 57], perhaps by interfering with NF-kB signaling [2]. Paralleling our results from LGG, other Lactobacilli also produce indoles and indole derivatives which reduce TNF-α, IL-1β and other pro-inflammatory cytokines as well as alter IL-22 signaling [14, 81].

Our results extend previous findings showing several probiotic *Lactobacillus* species other than LGG modestly inhibiting *P. acnes* growth in vitro [82]. The dramatic inhibitory effect of LGG on *P. acnes* growth may be mediated by LGG metabolites, LGG-induced inhibition of bacterial adhesion, or LGG-induced acidification of the lumen [83], and can be translated in vivo as indicated by LGG exclusion of *P. acnes* and *T. sanguinis* in the gut lumen. It will be interesting to determine in future work the mechanisms underlying LGG competitive exclusion and/or growth inhibition of commensal bacteria with pathobiont potential.

## Conclusions

The main findings of this study are that (1) *P. acnes* and *T. sanguinis abundances* are competitively inhibited by LGG, (2) the fecal metabolome of pathobionts is markedly altered by LGG, and (3) the inflammatory tone of the intestinal mucosa can be modified by LGG. An ancillary finding indicates that extraction of metabolites from feces is optimal when the volume of the extraction buffer is normalized to fecal sample abundance prior to analysis. While PTS is not particularly representative of naturally-found gut microbiomes, this study has been narrowly focused on metabolomic changes of these pathobionts/pathogens as a function of Lactobacillus administration. There is a need for additional studies to address the generalizability of these findings.

## Materials and methods

### Mice

For the first series of experiments involving optimization of metabolite extraction and normalization (Fig. 1a), eight-week old female and male C57BL/6 germ-free (GF) mice were purchased from Charles Rivers Laboratories (Wilmington, MA, USA). Following arrival, GF mice (*n =* 3) were unpacked and housed singly in autoclaved gnotobiotic cages on ventilated racks (Allentown, PA) at a temperature-controlled gnotobiotic facility at Rutgers New Jersey Medical School. GF mice were maintained on a 12:12 light-dark cycle and fed an autoclaved non-purified diet throughout the study (Supplemental Table 1A).

For the second set of experiments, eight-week old female C57BL/6 GF mice (*n =* 10) were purchased from Charles Rivers Laboratories. These mice were shipped in a sterilized GF container containing an autoclaved, non-purified diet and water. All GF mice were then maintained on a 12:12 light-dark cycle and given ad libitum access to a double-irradiated, purified diet (Research Diet, New Brunswick, NJ) (Supplemental Table 1B) and autoclaved water throughout the study. On designated days, feces were collected from 08:00 AM – 10:00 AM. Animal procedures and protocols were conducted in accordance with the Rutgers University Institutional Animal Care and Use Committee.

### Administration of PBS and LGG

*Lactobacillus rhamnosus* GG (LGG) (ATCC 53103) was purchased from American Tissue Culture Collection (ATCC, Manassas, Virginia, USA). LGG was cultured at 37 °C in MRS broth (Fisher Scientific, Pittsburgh, PA, USA) following the manufacturer’s protocol. The cultured broth was then centrifuged at 1200 g for 10 min to pellet the LGG. On Day 0 (Fig. 2a), a cohort of GF mice were gavaged with PBS, and another cohort with 200 μl of LGG (10^8^ CFU/ml) resuspended in the same PBS solution. The *P. acnes, T. sanguinis,* and *S. aureus* were identified from fecal samples of the PBS mice through bacterial 16S sequencing. Since *P. acnes* was virtually eliminated by LGG in vivo, we then tested LGG inhibitory effects on *P. acnes* (ATCC 51277) in vitro.

### Fecal collection and DNA extraction

Mice were placed in an empty autoclaved glass beaker and one fresh fecal pellet was transferred to a sterile 2 ml round-bottom tube (Nalgene, Rochester, NY) on dry ice and stored at − 80 °C until further analysis. For DNA extraction, lysis buffer was adjusted by fecal weight and up to 800 μl was added per bead-containing tube following the manufacturer’s instructions using Purelink Microbiome DNA Purification Kit (Invitrogen, Carlsbad, CA, USA). Samples were homogenized (30 s) in a bead homogenizer (Thermo Fisher, Waltham, MA) and centrifuged at 14000 g (5 min), and the supernatants were collected in a 1.5 mL microcentrifuge tube (Eppendorf, Hauppauge, NY). Samples were transferred to a spin column for purification and eluted in 100 μl elution buffer (Invitrogen by Thermo Fisher). DNA concentrations were determined by spectrophotometry (Nanodrop, Wilmington, DL). The purified DNA was used for bacterial 16S rRNA qPCR to monitor potential contamination. For bacterial 16S rRNA gene sequencing, DNA of feces from GF mice was extracted using QIAamp DNA Stool Mini kit following the manufacturer’s instructions (Qiagen Ltd., Strasse, Germany) for quality control.

### 16S rRNA gene sequencing analysis

Sequencing of the amplicon libraries was carried out using Ion Torrent™ Personal Genome Machine™ (PGM) system (PrimBio Research Institute, Philadelphia, PA) and the Oxford Nanopore MinION platform [84]. In order to calculate downstream diversity measures (alpha and beta diversity indices), 16S rRNA Operational Taxonomic Units (OTUs) were determined at ≥97% sequence homology.

### Quantitative real-time PCR (qPCR)

LGG and *P. acnes* was quantified in fecal samples by quantitative real-time PCR using Agilent AriaMx Real-time PCR system. The following primers: LGG forward, 5′-CGCCCTTAACAGCAGTCTTC-3′, reverse, 5′-GCCCTCCGTATGCTTAAACC-3′ and *P.acnes* forward, 5′-ATACGTAGGGTGCGAGCGTTGTCC-3′, reverse, 5′-TGGTGTTCCTCCTGATATCTGCGC-3′ were used. The qPCR reaction was performed using SYBR Green (Thermo Fisher) as follows: 95 °C for 5 min, followed by 45 cycles at 95 °C for 10 s, 65 °C for 15 s, and 72 °C for 15 s, and a final extension at 95 °C for 5 min, 65 °C for 1 min, and 98 °C for 30 s. For 16S rRNA quantitative PCR, primers that amplify gene encoding 16S rRNA from all bacterial groups were employed in the amplification reaction: 16S rRNA forward 5′-AGAGTTTGAT CCTGGCTCAG-3′ and reverse 5′-GACGGGCGGT GWGTRCA-3′. No significant bacterial amplicon was detected in pre-inoculation fecal samples, i.e., under GF condition, when analyzed by qPCR using universal primers to amplify bacterial 16S rRNA gene [85]. Here, the average Cq of fecal 16S rRNA was 30.8 + 0.4 before colonization, then 14.8 + 0.2 for PTS and 14.1 + 0.2 for LTS 5 days after colonization, consistent with studies showing absence and presence of bacteria [85].

### Extraction of metabolites and normalization of results

Three normalization procedures were experimentally evaluated: (1) no normalization (using raw feature data), (2) “Post-LC-MS” and (3) “Pre-LC-MS” normalization, as depicted in Fig. 1 (a and d). Frozen feces were weighed on a calibrated scale and placed on ice. The polar metabolites were extracted with a mixture of acetonitrile/methanol/water (40:40:20) containing 0.1 M formic acid (extraction buffer). For “Post-LC-MS” (Fig. 1a), equal volumes (500 μl) of the extraction buffer were added to all fecal samples regardless of the fecal weight, and the resulting metabolite level was normalized by fecal weight post LC-MS data acquisition. For “Pre-LC-MS” (Fig. 1d, normalization prior to LC-MS data acquisition), the volume of extraction buffer was adjusted by fecal weight prior to the extraction procedure. After addition of the extraction buffer to the feces, samples were then sonicated (4 °C, 30 s) and centrifuged (17,000 g, 2 min). After centrifugation, the supernatant collected (150 μl) was diluted by four-fold with the extraction buffer prior to LC-MS analysis.

### LC-MS metabolomics analysis and metabolites identification

The LC-MS analysis was performed on hydrophilic interaction chromatography coupled with electrospray ionization to the Q Exactive PLUS hybrid quadrupole-orbitrap mass spectrometer (Thermo Scientific) as previously described [86]. The LC separation was performed on a XBridge BEH Amide column (150 mm × 2.1 mm, 2.5 μm particle size, Waters, Milford, MA) using a gradient of solvent A (95%/5% H_2_O/ acetonitrile with 20 mM ammonium acetate and 20 mM ammonium hydroxide, pH 9.4), and solvent B (20%/80% H_2_O/ acetonitrile with 20 mM ammonium acetate and 20 mM ammonium hydroxide, pH 9.4). The gradient was 0 min, 100% B; 3 min, 100% B; 3.2 min, 90% B; 6.2 min, 90% B; 6.5 min, 80% B; 10.5 min, 80% B; 10.7 min, 70% B; 13.5 min, 70% B; 13.7 min, 45% B; 16 min, 45% B; 16.5 min, 100% B; 22 min, 100% B. The flow rate was 300 μL/min. Injection volume was 5 μL and column temperature 25 °C. Each sample was analyzed twice in both negative and positive ionization mode with a resolution of 70,000 at m/z 200. The automatic gain control target was 3 × 10^6^. The maximum injection time was 50 ms. Scan range was 75–1000. The MS2 spectra were collected from pooled samples under ddMS2 mode. The targeted metabolite data analysis was performed in MAVEN [87]. The compound identification was based on the accurate mass and the retention time learned from in-house chemical collection which includes 344 metabolites. The untargeted analysis was performed in Compound Discoverer (Thermo Scientific).

### Autoaggregation and coaggregation

*Propionibacterium acnes* (ATCC 51277) was purchased from American Tissue Culture Collection (ATCC, Manassas, Virginia, USA). *P. acnes* was cultured in AnaeroGRO Chopped Meat Glucose Broth (Hardy Diagnostics, Catalog No. AG19H) at 37 °C in an anerobic chamber. For autoaggregation experiments, *P. acnes* and LGG were harvested, washed with PBS, and re-suspended in 4 mL of blank meat broth respectively. The suspensions were then vortexed and incubated at room temperature for 48 h. The autoaggregation percentage was determined using the following equation: Autoaggregation (%) = [(A_0_ − A_t_)/A_0_] × 100, where A_0_ represents the absorbance (OD_600_) at time t = 0 h and A_t_ represents the absorbance (OD_600_) at time t = 48 h. For coaggregation experiments, equal volumes (2 mL) each of *P. acnes* and LGG were prepared, mixed by vortexing, incubated at room temperature for 48 h then absorbance (OD_600_) values were measured. The coaggregation percentage was determined using the following equation: Coaggregation (%) = [{(A_*P. acnes*_ + A_LGG_)/2 – A_*P. acnes* + LGG_}/ {(A_*P. acnes*_ + A_LGG_)/2}] × 100, Where A_*P. acnes*_ and A_LGG_ represents the absorbance (OD_600_) of *P. acnes* and LGG respectively at time t = 48 h as mentioned in the autoaggregation test and A_*P. acnes* + LGG_ represents the absorbance (OD_600_) of *P. acnes* and LGG combined and incubated for 48 h.

All tests for autoaggregation and coaggregation was repeated four times and the data were represented as mean ± SEM. The zone of clearance was measured as the distance from the meniscus of the solution to the end of a clear solution.

### Collection and analysis of intestinal tissues for expression of immune-related genes

At the end of the experiment, mice were anesthetized intraperitoneally (0.2 ml/100 g body weight) with ketamine (20 mg/ml) and xylazine (2.5 mg/ml). The intestine was quickly removed and divided into three segments: proximal (~ 18 cm) and distal (~ 18 cm) small intestine and colon (~ 6 cm). Segments were rinsed with ice-cold Dulbecco’s phosphate-buffered saline (DPBS; Life Technologies, Carlsbad, CA, USA), and opened longitudinally in fresh ice-cold DPBS to expose the mucosa. These were then placed on the surface of an ice-cold petri dish and the entire mucosa was scraped off with precooled microscopic slides. Mucosal scrapes (*n =* 5) were stored in 1.5 ml clean Eppendorf tubes at − 70 °C until analysis.

The intestinal tissue mucosa was thawed on ice, homogenized using the Bullet Blender centrifuge (Next Advance, Atkinson, NH, USA) and total RNA was isolated from the intestinal mucosa using RNeasy Mini Kit (Qiagen, CA, USA). cDNA was synthesized using Bio-Rad iScript™ RT Supermix (Bio-Rad, Hercules, CA). The screening of immune-related gene expression was performed by qPCR using SYBR Green Master Mix (ThermoFisher, Waltham, MA, USA) and primers of target genes are listed in Supplemental Table 2. Gene expression was normalized to the housekeeping gene, β-*actin*.

### Statistical analysis

Data are presented as mean ± SEM, (*n =* 5 except for data obtained for non-normalized and post LC-MS normalized). The bacterial OTUs (operational taxonomic units) using 16S profiling of Ion Torrent PGM platform were used for the calculation of diversity dissimilarities [88]. Beta diversity of distance metrics were estimated UniFrac distances and followed by Bray-Curtis analysis to represent taxon-based difference within bacterial communities from different sample groups. The calculated data was defined by clustering using Principal Coordinate Analysis (PCoA) to assess patterns in the different sample groups. For metabolites analysis, Principal component analysis (PCA) and partial least squares discriminant analysis (PLS-DA) were performed to visualize differences in patterns of fecal metabolite levels between PTS and LTS or between the pre-gavage GF condition and each of the two groups [89]. We then calculated the VIP (variable’s importance (influence) on the projection) scores of each metabolite based on the PLS-DA [90]. The VIP summarizes the contribution of a specific metabolite associated with either PTS or LTS colonization. A VIP score cut-off of > 1.2 was used as a variable selection based on discriminatory power, since VIP > 1 is most influential for the model shown [48].

Student’s t-test was performed to elicit statistically significant differences of the two sample groups in metabolites data analysis between GF and PTS, or PTS and LTS. For the qPCR data in Fig. 2, statistical significances were assessed by two-way ANOVA and Tukey’s post-hoc test. The metabolomics datasets were analyzed by pattern recognition methods using MetaboAnalyst 4.0. Multivariate statistical analysis was conducted using MetaboAnalyst 4.0 PCA and PLS-DA were performed to find difference between metabolite profiles [47].

## Supplementary Information

**Additional file 1: Supplementary Table 1A**. Nutrient composition of autoclavable rodent pellets used in experiments optimizing the metabolite extraction procedure. **Supplementary Table 1B**. Nutrient composition of the diet used in the LTS and PTS experiment. **Supplementary Table 2**. Primer sequences used in RT-qPCR of mouse genes. **Supplementary Table 3A**. VIP scores and regulation of fecal metabolites in LTS compared to PTS mice. **Supplementary Table 3B**. VIP scores and regulation of fecal metabolites in GF compared to PTS mice. **Supplementary Table 3C**. VIP scores and regulation of fecal metabolites in GF compared to LTS mice. **Supplementary Table 3D**. Highly significant VIP metabolites that are up or downregulated with PTS relative to GF, with LTS relative to GF, and LTS relative to PTS fall into four categories: (1) those that are upregulated in both PTS and LTS, (2) downregulated in both PTS and LTS, (3) upregulated in PTS or downregulated in LTS, and (4) upregulated in LTS. **Supplementary Table 4**. Accurate m/z and relative intensity values of peaks in the ms2 spectra of unidentified metabolites.

**Additional file 2: Supplementary Fig. 1**. Experimental design and clinical parameters (A) Schematic diagram of experimental plan (B) Body weights as a function of age of mice before and after gavage with PTS or LTS (C) Diet consumption by mice over the course of the study. **Supplementary Fig. 2**. Abundance of pathobionts when coassociated with LGG. Propionibacterium (A), Lactobacillus (B), Turicibacter (C), and Staphylococcus (D) abundance was determined in the feces from PTS (*n* = 5) and LTS (*n =* 5) mice by 16S amplicon profiling. **Supplementary Fig. 3.** Unsupervised principal component analysis (PCA) of fecal metabolites from GF, PTS, and LTS mice. **Supplementary Fig. 4**. Accurate mass and MS2 spectra of unidentified metabolites. **Supplementary Fig. 5**. Relative mRNA expression of intestinal immunerelated genes in mucosa of proximal small intestine in GF mice associated PTS or LTS.

## Data Availability

The 16S rRNA sequencing data generated in this study are deposited in SRA under accession number: PRJNA655706. The metabolomics data generated in this study are deposited in the MassIVE database under project MSV000085927.

## Abbreviations

AHR: Aryl hydrocarbon receptor
DSS: Dextran sodium sulfate
GF: Germ-free
IL-1: Interleukin 1
LGG: *Lactobacillus rhamnosus* GG
LC-MS: Liquid chromatography-mass spectrometry
LTS: *Lactobacillus rhamnosus* GG plus *Propionibacterium acnes, Turicibacter sanguinis,* and *Staphylococcus aureus*
OTU: Operational taxonomic unit
PCA: Principal component analysis
PCoA: Principal coordinate analysis
PLS-DA: Partial least squares discriminant analysis
PTS: *Propionibacterium acnes, Turicibacter sanguinis,* and *Staphylococcus aureus*
TNF-α: Tumor necrosis factor-alpha
VIP: Variable’s importance in the projection
