# Supplementary material for: Lactobacillus rhamnosus GG modifies the metabolome of pathobionts in gnotobiotic mice
Source: BMC Microbiol. 2021 Jun 3;21:165. doi: 10.1186/s12866-021-02178-2 (PMC8176599; doi:10.1186/s12866-021-02178-2)
Supplement: Supplementary file 1 — Additional file 1: Supplementary Table 1A. Nutrient composition of autoclavable rodent pellets used in experiments optimizing the metabolite extraction procedure. Supplementary Table 1B. Nutrient composition of the diet used in the LTS and PTS experiment. Supplementary Table 2. Primer sequences used in RT-qPCR of mouse genes. Supplementary Table 3A. VIP scores and regulation of fecal metabolites in LTS compared to PTS mice. Supplementary Table 3B. VIP scores and regulation of fecal metabolites in GF compared to PTS mice. Supplementary Table 3C. VIP scores and regulation of fecal metabolites in GF compared to LTS mice. Supplementary Table 3D. Highly significant VIP metabolites that are up or downregulated with PTS relative to GF, with LTS relative to GF, and LTS relative to PTS fall into four categories: (1) those that are upregulated in both PTS and LTS, (2) downregulated in both PTS and LTS, (3) upregulated in PTS or downregulated in LTS, and (4) upregulated in LTS. Supplementary Table 4. Accurate m/z and relative intensity values of peaks in the ms2 spectra of unidentified metabolites. [file 12866_2021_2178_MOESM1_ESM.pdf]

Supplementary Tables.

Lactobacillus rhamnosus GG modifies the metabolome of pathobionts in gnotobiotic mice

Jinhee Kim+, Iyshwarya Balasubramanian+, Sheila Bandyopadhyay, Ian Nadler, Rajbir Singh, Danielle Harlan, Amanda Bumber, Yuling He, Lee J. Kerkhof, Nan Gao, Xiaoyang Su, Ronaldo P. Ferraris

+, equal contribution

Supplementary Table 1A. Nutrient composition of autoclavable rodent pellets used in experiments optimizing the metabolite extraction procedure.

	Chow diet (LabDiet® JL 5KA1)
**Macronutrients**	**Total % kcal**
Proteins	22.2
Carbohydrates	61.6
Fats (ether extract)	16.2
Total	100
**Relevant amino acid**	
**Tryptophan %**	0.23

Calculated values provided by the manufacturer, LabDiet®. Additional information may be obtained from http://jackson.jax.org/rs/444-BUH-304/images/LabDiet_5K52.pdf

Supplementary Table 1B. Nutrient composition of the diet used in the LTS and PTS experiment.

	L-Amino Acid Rodent diet (Research Diet A10021B Su-1.5V)	
**Macronutrients**	Total (% g)	Total (% kcal)
Proteins	17.0	17.7
Carbohydrates	68.5	70.6
Lipids	5.0	11.7
Total		100.0
kcal/gm	3.8	
**Ingredient**	**g/kg diet**	**Kcal**
**Protein**		
L-Amino Acids, total	170	680
L-Tryptophan	2	8
**Carbohydrate**		
Corn Starch	550.5	2202
Maltodextrin 10	125	500
Sucrose	8.5	34
**Fat**		
Corn Oil	50	450
**Fiber**		
Cellulose	50	0
**Mineral Mix S10001**	35	0
Sodium Bicarbonate	7.5	
**Vitamin Mix V10001 (10X Concentrated)**	1.5	6
Choline Bitartrate	2	0
**Dye**		
Blue dye	0.05	0
**Total**	1000.05	3838

This diet formulated by E. A. Ulman, Ph.D., Research Diets, Inc., 5/3/94 and 11/12/99.

Supplementary Table 2. Primer sequences used in RT-qPCR of mouse genes

Gene	Forward primer (5' to 3')	Reverse primer (5' to 3')
Il1α	ACGTCAAGCAACGGGAAGAT	AAGGTGCTGATCTGGGTTGG
Il1β	CCTTGGGCCTCAAAGGAAAG	TCTTCTTCTTTGGGTATTGCTTGG
Tnfα	ACAAGCCTGTAGCCCACGTC	CTTTGAGATCCATGCCGTTG
Il22	GTGACGACCAGAACATCCAGA	AAACAGCAGGTCCAGTTCCC
Il22r1	ATTTGGGAGTGGAGAGGATG	ACAGCTGCCCTGCTTCTTAT
Amphiregulin (Areg)	CGGCATCGTTATCACAGTGC	TGCACAGTCCCGTTTTCTTG
Il10	TCTCCCCTGTGAAAATAAGAGCA	TTCATGGCCTTGTAGACACCT
Il12α	CGCAGCACTTCAGAATCACA	CGCAGAGTCTCGCCATTATG
Il6	AAATTTCCTCTGGTCTTCTGGAG	CGCACTAGGTTTGCCGAGTA
Il5	AAGGATGCTTCTGCACTTGA	ACACCAAGGAACTCTTGCA
Ifnγ	GCGTCATTGAATCACACCTGA	CTGGACCTGTGGGTTGTTGA
Il13	CGGTGCCAAGATCTGTGTCT	ACACTCCATACCATGCTGCC
Il17	ATCCCTCAAAGCTCAGCGTGTC	GGGTCTTCATTGCGGTGGAGAG
Gata3	GAGGAGGAACGCTAATGGGG	CGGGTCTGGATGCCTTCTTT
Zo1	GGGAGGGTCAAATGAAGACA	GGCATTCCTGCTGGTTACAT
Cldn4	GGCTGCTTTGCTGCAACTGTC	GAGCCGTGGCACCTTACACG
Vegfa	AAAGGCTTCAGTGTGGTCTGAGAG	GGTTGGAACCGGCATCTTTATC
Cyp1a1	CTCTTCCCTGGATGCCTTCAA	GGATGTGGCCCTTCTCAAATG
β-actin	TTGTTACCAACTGGGACGACATGG	CTCGGGTGTTGAAGGTCTCAAACA

Supplementary Table 3A. VIP scores and regulation of fecal metabolites in LTS compared to PTS mice*.

Rank	Metabolites	VIP score	Upregulated in LTS	Downregulated in LTS
1	N-Acetylputrescine_Pos	2.4135	O	
2	N-acetyl-glutamine_Neg	2.4023	O	
3	N-acetyl-glutamine_Pos	2.3843	O	
4	Sorbitol_Neg	2.3584		O
5	Guanine_Neg	2.2516		O
6	Levulinic Acid_Neg	1.9817	O	
7	Ornithine_Neg	1.9768		O
8	Acetyl leucine_Neg	1.9448	O	
9	Carnitine_Pos	1.9265	O	
10	Guanine_Pos	1.9016		O
11	Pyroglutamate_Neg	1.8805	O	
12	Ornithine_Pos	1.8693		O
13	Citrulline_Pos	1.8571		O
14	Histamine_Pos	1.8032	O	
15	Ribitol_Neg	1.8024	O	
16	Acetyl-arginine_Pos	1.7218		O
17	Indolelactic acid_Neg	1.7069	O	
18	Guanidinoacetate_Pos	1.6693		O
19	Nicotinate_Neg	1.632	O	
20	2 2-dimethylsuccinic acid_Pos	1.5725	O	
21	Citrulline_Neg	1.5516		O
22	Hypoxanthine_Neg	1.5192	O	
23	Aspartate_Neg	1.5182	O	
24	Adenine_Pos	1.4747		O
25	Cystine_Pos	1.4368		O
26	Adenine_Neg	1.3983		O
27	Xanthine_Neg	1.3591	O	
28	Lysine_Pos	1.3445		O
29	Pyruvate_Neg	1.338		O
30	Isoleucine_Pos	1.3325		O
31	Creatinine_Pos	1.3292	O	
32	Stearic acid_Neg	1.3119	O	
33	Hypoxanthine_Pos	1.2866	O	
34	Aspartate_Pos	1.2728	O	
35	Proline_Pos	1.2688		O
36	Acetyl-Phenylalanine_Neg	1.2447	O	
37	Urocanic acid_Pos	1.2288		O
38	Creatinine_Neg	1.2258	O	

39	Isoleucine_Neg	1.2094		O

Supplementary Table 3B. VIP scores and regulation of fecal metabolites in GF compared to PTS mice*.

Rank	Metabolites	VIP score	Upregulated in PTS	Downregulated in PTS
1	Ornithine_Pos	1.9391	O	
2	Betaine_Pos	1.9057	O	
3	Carnitine_Pos	1.869	O	
4	Citrulline_Neg	1.8386	O	
5	Ornithine_Neg	1.7347	O	
6	Glutamate_Pos	1.706	O	
7	Spermine_Pos	1.6971	O	
8	Quinic acid_Neg	1.6813		O
9	Citrulline_Pos	1.6397	O	
10	Guanine_Neg	1.6342	O	
11	NG-dimethyl-L-arginine_Pos	1.5952	O	
12	Ribitol_Neg	1.5864		O
13	Biotin_Pos	1.579		O
14	Biotin_Neg	1.5682		O
15	5-Methylcytosine_Pos	1.5563		O
16	Glutamate_Neg	1.5462	O	
17	Histidine_Pos	1.5044	O	
18	Cytosine_Pos	1.4986		O
19	Aspartate_Pos	1.4866	O	
20	Nicotinamide riboside_Pos	1.4604	O	
21	Creatinine_Neg	1.4354		O
22	Nicotinate_Neg	1.435	O	
23	Sorbitol_Neg	1.427		O
24	Adenine_Pos	1.4226	O	
25	Deoxyinosine_Pos	1.4176		O
26	Adenine_Neg	1.4095	O	
27	Creatinine_Pos	1.3964		O
28	Arginine_Neg	1.389		O
29	Arginine_Pos	1.3792		O
30	Histamine_Pos	1.3588	O	
31	Inositol_Neg	1.3464		O
32	Guanosine_Neg	1.3428		O
33	Thymidine_Neg	1.3417		O
34	Inosine_Neg	1.3415		O
35	Guanidinoacetate_Pos	1.3369	O	
36	Glutaconic acid_Neg	1.3359		O
37	Deoxyguanosine_Pos	1.3354		O

38	D-Pantethine_Pos	1.3325	O	
39	Cytidine_Pos	1.3285		O
40	1-Methylimidazole acetic acid_Pos	1.3104	O	
41	Uridine_Neg	1.3066		O
42	Deoxyinosine_Neg	1.2975		O
43	Citrate_Neg	1.2937		O
44	Guanosine_Pos	1.2721		O
45	Cytidine_Neg	1.2693		O
46	Isoleucine_Pos	1.2606	O	
47	Deoxyguanosine_Neg	1.2471		O
48	Threonic acid_Neg	1.2177		O
49	Proline_Pos	1.2134	O	

Supplementary Table 3C. VIP scores and regulation of fecal metabolites in GF compared to LTS mice*.

Rank	Metabolites	VIP score	Upregulated in LTS	Downregulated in LTS
1	Carnitine_Pos	1.8916	O	
2	N-Acetylputrescine_Pos	1.8861	O	
3	Sorbitol_Neg	1.8545		O
4	N-acetyl-glutamine_Pos	1.8438	O	
5	N-acetyl-glutamine_Neg	1.8231	O	
6	Betaine_Pos	1.8222	O	
7	2 2-dimethylsuccinic acid_Pos	1.8195	O	
8	Glutamate_Pos	1.6922	O	
9	Levulinic Acid_Neg	1.68	O	
10	Thymidine_Neg	1.6798		O
11	Aspartate_Pos	1.6538	O	
12	Pyruvate_Neg	1.6179		O
13	Histamine_Pos	1.6126	O	
14	Glutamate_Neg	1.6063	O	
15	Nicotinate_Neg	1.5997	O	
16	Guanine_Pos	1.5977		O
17	Acetyl leucine_Neg	1.5951	O	
18	Quinic acid_Neg	1.5884		O
19	Deoxyinosine_Pos	1.585		O
20	5-Methylcytosine_Pos	1.5568		O
21	Histidine_Pos	1.5319	O	
22	N-acetyl-glutamate_Neg	1.5278	O	
23	Biotin_Neg	1.5184		O
24	Cytosine_Pos	1.5149		O
25	NG-dimethyl-L-arginine_Pos	1.5023	O	
26	Biotin_Pos	1.4812		O
27	Thymine_Pos	1.4809		O
28	Thymine_Neg	1.4606	O	
29	Citrulline_Neg	1.4601	O	
30	Glucosamine_Pos	1.434		O
31	Pyroglutamate_Neg	1.4283	O	
32	Nicotinamide riboside_Pos	1.4236	O	
33	Spermine_Pos	1.4184	O	
34	Guanosine_Neg	1.3791		O
35	Leucic acid_Neg	1.3596	O	
36	Deoxyguanosine_Pos	1.3534		O
37	Deoxyinosine_Neg	1.3418		O
38	Uridine_Neg	1.3411		O

39	Guanosine_Pos	1.3262		O
40	Indolelactic acid_Neg	1.3163	O	
41	5- Methylthioadenosine_Neg	1.2986		O
42	Cytidine_Neg	1.2939		O
43	Citrulline_Pos	1.2869	O	
44	Ornithine_Pos	1.2406	O	
45	Cytidine_Pos	1.2365		O
46	Trimethylamine oxide_Pos	1.2335		O
47	Nicotinate_Pos	1.2324	O	
48	Serine_Neg	1.221		O

Supplementary Table 3D. Highly significant VIP metabolites that are up or downregulated with PTS relative to GF, with LTS relative to GF, and LTS relative to PTS fall into four categories: (1) those that are upregulated in both PTS and LTS, (2) downregulated in both PTS and LTS, (3) upregulated in PTS or downregulated in LTS, and (4) upregulated in LTS*.

METABOLITE, MODE	VIP rank, GF vs PTS	VIP rank, GF vs LTS	VIP rank, PTS vs LTS	statistical comparison**
**1. upregulated in both PTS and LTS compared to GF**				
carnitine, pos	3, high PTS	1, high LTS	9, high LTS	LTS>>>PTS>>>GF
betaine, pos	2, high PTS	6, high LTS	45, low LTS	PTS>>>LTS>>>GF
glutamate, neg	16, high PTS	14, high LTS	not ranked	LTS=PTS>>>GF
glutamate, pos	6, high PTS	8, high LTS	not ranked	LTS=PTS>>GF
aspartate, neg	not ranked	not ranked	23, high LTS	LTS>>PTS=GF
aspartate, pos	19, high PTS	11, high LTS	34, high LTS	LTS>PTS>>>GF
nicotinate, pos	67, high PTS	47, high LTS	not ranked	LTS>PTS>>>GF
nicotinate, neg	22, high PTS	15, high LTS	19, high LTS	LTS>>>PTS>>>GF
NG-dimethyl-L-arginine, pos	11, high PTS	25, high LTS	not ranked	LTS=PTS>>> GF
histidine, pos	17, high PTS	21, high LTS	not ranked	LTS=PTS>>>GF
N-acetyl-glutamate, neg	not ranked	22, high LTS	52, LTS=PTS	LTS=PTS>>>GF
spermine, pos	7, high PTS	33, high LTS	not ranked	PTS=LTS>>>GF
**2. downregulated in both PTS and LTS compared to GF**				
thymidine, neg	33, low PTS	10, low LTS	49, low LTS	GF>>>PTS>LTS
quinic acid, neg	8, low PTS	18, low LTS	not ranked	GF>>>PTS=LTS
biotin, pos	13, low PTS	26, low LTS	not ranked	GF>>>PTS=LTS
biotin, neg	14, low PTS	23, low LTS	not ranked	GF>>>PTS=LTS
5-methylcytosine, pos	15, low PTS	20, low LTS	not ranked	GF>>>PTS=LTS
inosine, neg	34, low PTS	62, low LTS	53, low LTS	GF>>>PTS=LTS
guanosine, pos	44, low PTS	39, low LTS	not ranked	GF>>>PTS=LTS
guanosine, neg	32, low PTS	34, low LTS	not ranked	GF>>>PTS=LTS
**3. upregulated in PTS or downregulated in LTS**				
guanine, pos	not ranked	16, low LTS	10, low LTS	GF=PTS>>>LTS
guanine, neg	10, high PTS	not ranked	5, low LTS	PTS>>>GF=LTS
ornithine, neg	5, high PTS	not ranked	7, low LTS	PTS>>>LTS>GF
ornithine, pos	1, high PTS	44, high LTS	12, low LTS	PTS>>>LTS>GF
citrulline, pos	4, high PTS	29, high LTS	13, low LTS	PTS>>>LTS>GF
pyruvate, neg	72	12, low LTS	29, low LTS	GF=PTS >>LTS
sorbitol, neg	23, low PTS	3, low LTS	4, low LTS	GF>>>PTS>>>LTS
isoleucine, pos	46, high PTS	30, low LTS	39, low LTS	PTS>LTS>GF

4. upregulated in LTS				
N-acetyl-putrescine, pos	not ranked	2, high LTS	1, high LTS	LTS>>>PTS>GF
N-acetyl-glutamine, neg	not ranked	4, high LTS	2, high LTS	LTS>>>PTS=GF
N-acetyl-glutamine, pos	not ranked	5, high LTS	3, high LTS	LTS>>>PTS=GF
acetyl leucine, neg	high PTS	17, high LTS	8, high LTS	LTS>>>PTS>>GF
pyroglutamate, neg	high PTS	31, high LTS	11, high LTS	LTS>>>PTS>GF
ribitol, neg	12, low PTS	not ranked	15, high LTS	LTS = GF>>>PTS
hypoxanthine, neg	not ranked	70, high LTS	22, high LTS	LTS>>>PTS>GF
indolelactic acid, neg	not ranked	40, high LTS	17, high LTS	LTS>>>PTS=GF
levulinic acid, neg	76, high PTS	9, high LTS	6, high LTS	LTS>>>PTS>>>GF
histamine, pos	30, high PTS	13, high LTS	14, high LTS	LTS>>>PTS>>>GF
2,2-dimethylsuccinic acid,pos	51, high PTS	7, high LTS	20, high LTS	LTS>>>PTS>>>GF

* GF = germ-free; LTS = *Propionibacterium acnes, Turicibacter sanguinis,* and *Staphylococcus aureus* plus *Lactobacillus rhamnosus* GG; PTS = *Propionibacterium acnes, Turicibacter sanguinis,* and *Staphylococcus aureus*; VIP = variable's influence on projection; (pos) and (neg) refer to the positive and negative mode of LC-MS analysis, respectively.

** number of >'s correspond to significance level, so > = p<0.05; >> = p<0.025; >>> = p<0.01

Supplementary Table 4. Accurate m/z and relative intensity values of peaks in the ms2 spectra of unidentified metabolites

Figure	Feature	m/z	Relative Intensity
Fig. 5E, Supplemental Fig. 4A	163.0607@3.68min	71.01384	0.4389
		85.02937	0.5183
		163.0614	0.7340
Fig. 5F, Supplemental Fig. 4C	357.1028@9.68min	57.03438	0.0374
		59.0136	0.0717
		69.03444	0.0034
		71.0138	0.2396
		72.993	0.0746
		73.02945	0.0316
		75.00867	0.9038
		83.01374	0.0396
		85.02937	0.1281
		87.00861	0.0224
		89.02429	0.0886
		95.01363	0.0064
		99.00866	0.0283
		101.0243	0.1398
		111.0086	0.0318
		113.0243	0.0610
		119.0347	0.0274
		129.0192	0.2190
		131.0352	0.0042
		141.0192	0.0237
		159.0298	0.0589
		221.066	0.0311
		357.1035	0.0862
Supplemental Fig. 4A	237.0973@2.25min	51.38644	0.4197
		70.09301	0.4748
		113.06578	0.5474
		119.14967	0.5467
Supplemental Fig. 4B	257.0772@9.08min	82.029778	0.3624
		84.045319	0.1364
		102.05617	0.0595
		109.07697	0.0677
		110.02458	0.0364
		113.07207	0.0869
		127.05127	0.1856
		128.03529	0.8771
		146.04614	0.0115
		169.09818	0.1414

		195.07753	0.0439
		213.08829	0.0642
		239.06747	0.0567
Supplemental Fig. 4B	287.0764@4.76min	65.03968	0.0468
		67.018929	0.0642
		69.0345	0.1702
		95.013687	0.0637
		95.050179	0.2849
		97.029625	0.3121
		105.03448	0.0830
		108.02132	0.0446
		109.02956	0.2438
		111.04471	0.0568
		113.02443	0.1095
		121.02934	0.2361
		123.04514	0.5428
		125.02436	0.2147
		143.03485	0.0954
		149.02458	0.2120
		153.01907	0.0852
		153.05618	0.1032
		167.03473	0.3604
		169.05081	0.1987
		199.06154	0.1014
		209.04472	0.0709
		239.0556	0.0597
		287.07657	0.2046
Supplemental Fig. 4C	340.1140@9.15min	82.029846	0.0557
		109.04054	0.0837
		114.05594	0.1298
		127.0512	0.9847
		137.07202	0.0417
		153.06703	0.0414
Supplemental Fig. 4D	288.192@6.16min	59.01371	1.0000
Supplemental Fig. 4D	201.0877@4.45min	51.297226	0.0158
		53.253372	0.0173
		69.648727	0.0161
		77.349976	0.0150
		88.040443	0.7645
		98.061676	0.0298
		112.04061	0.0621
		115.08768	0.6382
		128.95592	0.0185

		151.82323	0.0175
		159.07768	0.0431
