# Supplementary material for: Lactobacillus rhamnosus GG modifies the metabolome of pathobionts in gnotobiotic mice
Source: BMC Microbiol. 2021 Jun 3;21:165. doi: 10.1186/s12866-021-02178-2 (PMC8176599; doi:10.1186/s12866-021-02178-2)
Supplement: Supplementary file 2 — Additional file 2: Supplementary Fig. 1. Experimental design and clinical parameters (A) Schematic diagram of experimental plan (B) Body weights as a function of age of mice before and after gavage with PTS or LTS (C) Diet consumption by mice over the course of the study. Supplementary Fig. 2. Abundance of pathobionts when coassociated with LGG. Propionibacterium (A), Lactobacillus (B), Turicibacter (C), and Staphylococcus (D) abundance was determined in the feces from PTS (n = 5) and LTS (n = 5) mice by 16S amplicon profiling. Supplementary Fig. 3. Unsupervised principal component analysis (PCA) of fecal metabolites from GF, PTS, and LTS mice. Supplementary Fig. 4. Accurate mass and MS2 spectra of unidentified metabolites. Supplementary Fig. 5. Relative mRNA expression of intestinal immunerelated genes in mucosa of proximal small intestine in GF mice associated PTS or LTS. [file 12866_2021_2178_MOESM2_ESM.pdf]

Supplementary Figures.

Lactobacillus rhamnosus GG modifies the metabolome of pathobionts in gnotobiotic mice

Jinhee Kim+, Iyshwarya Balasubramanian+, Sheila Bandyopadhyay, Ian Nadler, Rajbir Singh, Danielle Harlan, Amanda Bumber, Yuling He, Lee J. Kerkhof, Nan Gao, Xiaoyang Su, Ronaldo P. Ferraris
+, equal contribution

Kim et al. Supplementary Figure 1

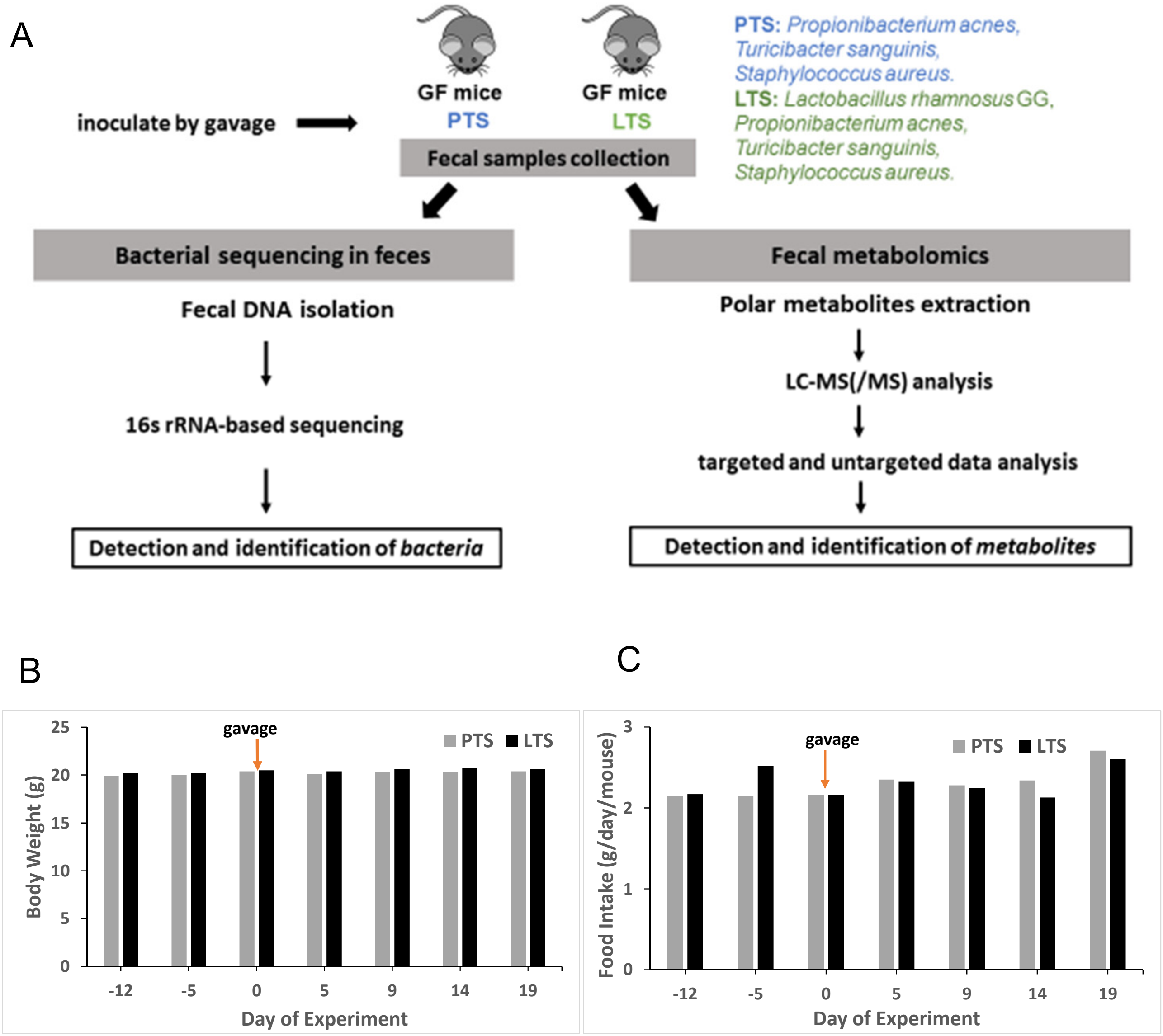

Supplementary Fig 1. Experimental design and clinical parameters (A) Schematic diagram of experimental plan (B) Body weights as a function of age of mice before and after gavage with PTS or LTS (C) Diet consumption by mice over the course of the study.

Kim et al. Supplementary Figure 2

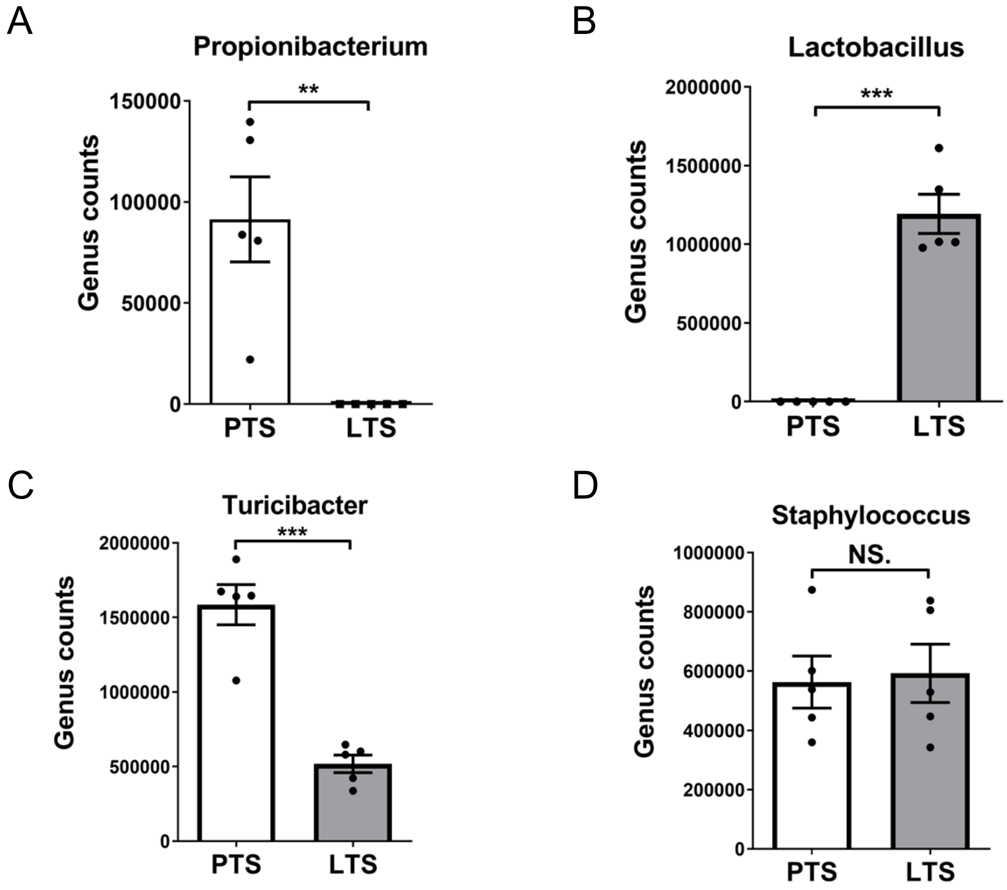

Supplementary Fig 2. Abundance of pathobionts when coassociated with LGG. Propionibacterium (A), Lactobacillus (B), Turicibacter (C), and Staphylococcus (D) abundance was determined in the feces from PTS (n=5) and LTS (n=5) mice by 16S amplicon profiling.

Kim et al. Supplementary Figure 3

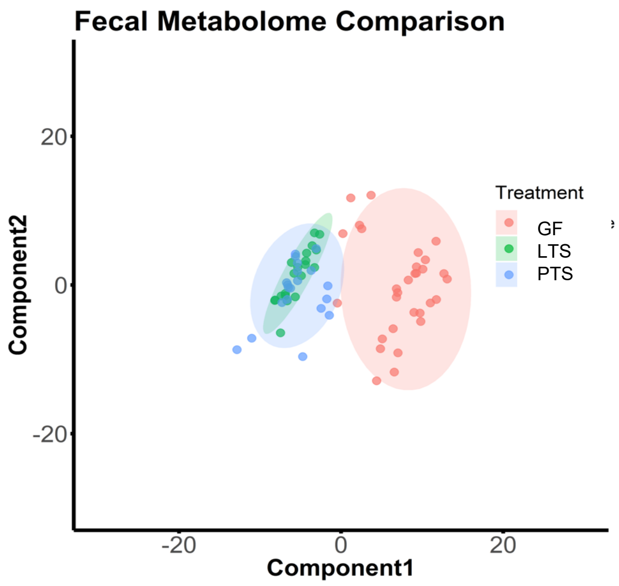

Supplementary Fig 3. Unsupervised principal component analysis (PCA) of fecal metabolites from GF, PTS, and LTS mice.

Kim et al. Supplementary Figure 4

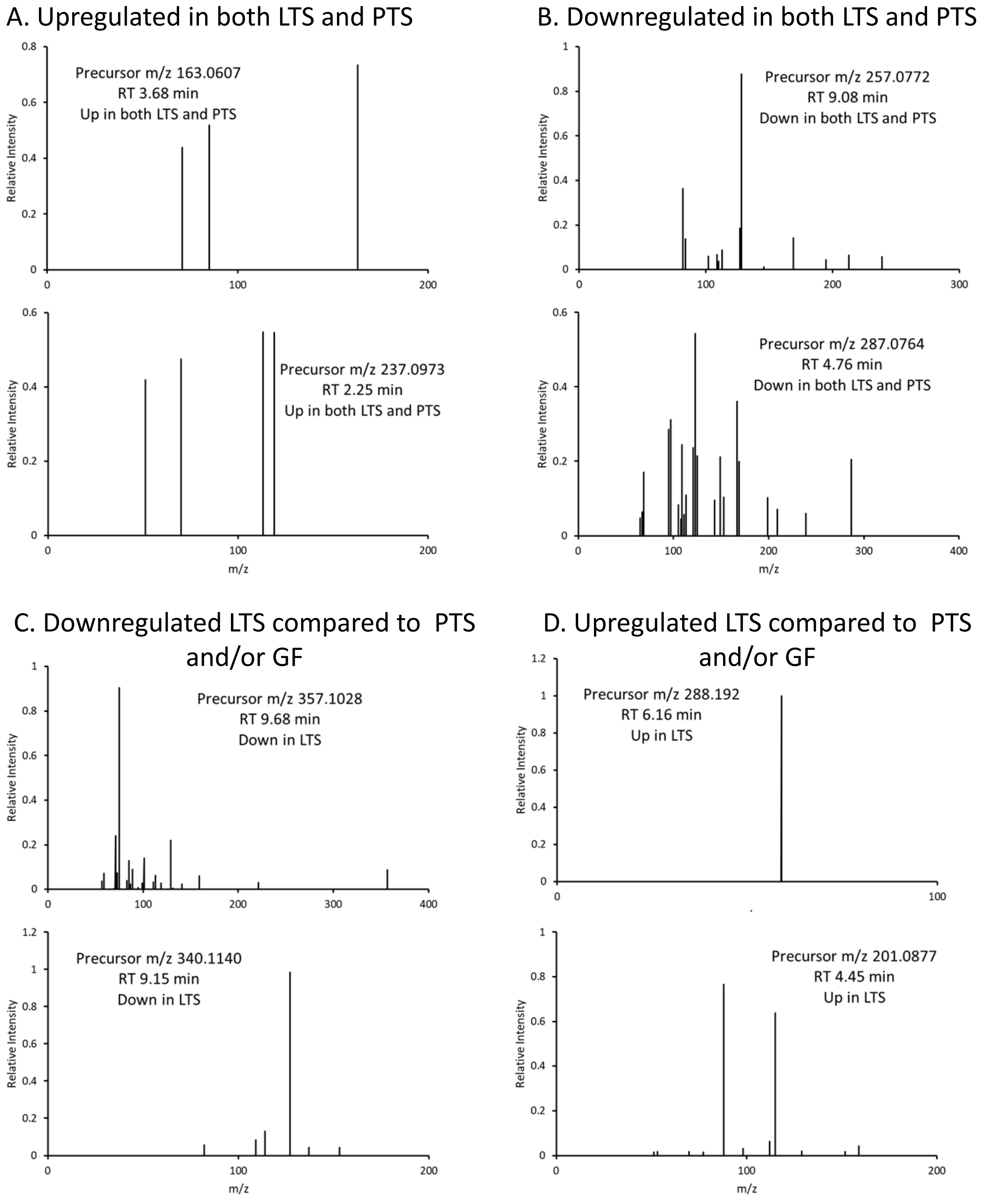

Supplementary Fig. 4. Accurate mass and MS^2 spectra of unidentified metabolites

Kim et al. Supplementary Figure 5

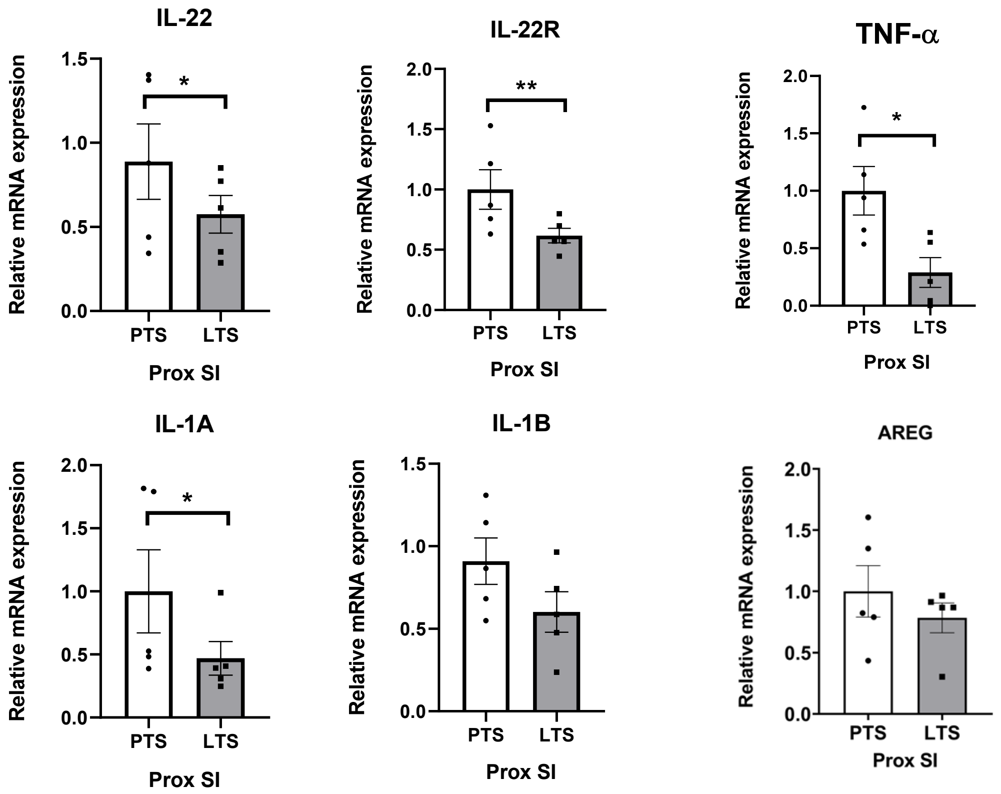

Supplementary Figure 5. Relative mRNA expression of intestinal immune-related genes in mucosa of proximal small intestine in GF mice associated PTS or LTS
